# Case of Hydrophobia, Successfully Treated by the Scutellaria Lateriflora

**Published:** 1820-06

**Authors:** 


FOR THE LONDON MEDICAL AND PHYSICAL JOURNAL.

### *Case of Hydrophobia, successfully treated by the Scutellaria Lateriflora.* By Dr. Fiske.

MRS. H—, belonging to this town, (Montague, Mass.) of a healthy constitution, twenty-four years of age, was bitten on the 5th day of July last by a puppy four months old, supposed by herself and her friends to be mad. The following were the marks he showed of rabies: On Saturday the 3d he refused his accustomed food; appeared stupid and sickly, head and ears hanging down, and showed no disposition for playfulness. On the 4th, still refused to eat; his eyes were red, dull, and full of tears, and his mouth covered with apparently tough and frothy slime. He frequently staggered and fell down; sometimes started up quick, and attempted to run, but could not get straight forward; took little notice of any thing; towards evening snapped at objects, but never barked. On Monday morning he became furious; run at every thing that came in his way, and attempted to bite; at length actually did bite the lady above mentioned on the ball of the thumb, making four incisions through the skin. He was then immediately killed. On the same day she sent for me, and I advised the immediate use of the scullcap; but, not having any on hand, I was only able to procure some of another person, which had been gathered two years before, and had lain exposed to the open air in a box, with directions to give it every other day, as prescribed by Dr. Thacher. She did so; and the wound healed in a few days, with no unpleasant symptoms. But, on the fifteenth day after the bite, she felt a slight pain or itching in the part bitten, which soon became a little elevated; and a circumscribed inflamed spot, about the size of a sixpence, arose, and extended over the cicatrix of one of the marks of the dog's teeth. Soon afterwards, she felt a fixed pain in the wrist, which extended to her elbow, and shortly increased and reached to her shoulder; wandering pains in her back and joints succeeded; she felt a painful and strange sensation in her head, and sometimes also a giddiness, so that she could not walk straight forwards. She now complained of lassitude, with stricture and heaviness in her breast, accompanied with difficulty of breathing.

On perceiving these alarming symptoms, I concluded that the plant had lost its virtues by age and exposure, and endeavoured to procure some of recent growth, which fortunately I obtained, and in blossom. Of this I ordered a strong decoction to be taken immediately, in doses of half-a-pint each, four times a-day; to be suspended every other day, and a table-spoonful of flowers of sulphur, in new milk, to be taken in its stead. For the greater precaution, I also punctured the bitten part, which discharged a little watery fluid, and applied to it the bruised leaves of the plant, which I renewed once every four or six hours.

On the sixteenth day, symptoms were but little abated, and her pulse somewhat depressed; but she had slept more quietly. The seventeenth, the pain, excepting in her wrist and head, had subsided; the bite had lost its redness, and she had slept still better. On the eighteenth, she said she felt quite well, excepting a little weakness. She kept the application on the wound two days longer, when it healed, and she left it off; but continued to take the decoction, though with diminished strength, three weeks longer; but has experienced no pain or unpleasant sensation since the eighteenth day, enjoying her usual good health, and going about her domestic labours as formerly.

The above symptoms were noted down at the time of their appearance. Mrs. H. is by no means of a nervous temperament, but resolute; and she followed my prescription with much confidence.*

*Montague, Massachusetts; August* 22, 1819.

* We adduce these cases in detail, in addition to our former observations on this subject, from the consideration, that more precise accounts than general statements are necessary in order to satisfy the enquiries of medical practitioners on so important a subject. These cases are the only ones which are detailed in a satisfactory manner; and, we must remark, they have more of the character of *tetanus* than of *hydrophobia*.—Edit.

